# New 675 nm Laser Device: The Innovative and Effective Non-Ablative Resurfacing Technique

**DOI:** 10.3390/medicina59071245

**Published:** 2023-07-04

**Authors:** Domenico Piccolo, Giuliana Crisman, Caterina Dianzani, Iris Zalaudek, Irene Fusco, Claudio Conforti

**Affiliations:** 1 Novea Skin Center, 67051 Avezzano, Italy; domenico.piccolo.skincenters@gmail.com (D.P.); giuliana.crisman@gmail.com (G.C.); 2 Plastic Surgery Unit, Section of Dermatology, Campus Biomedico University, 00128 Rome, Italy; c.dianzani@policlinicocampus.it; 3 Dermatology Clinic, University of Trieste, 34126 Trieste, Italy; izalaudek@units.it (I.Z.); claudioconforti@yahoo.com (C.C.); 4 El.En. Group, 50041 Calenzano, Italy

**Keywords:** Modified Fitzpatrick Wrinkles Scale, 675 nm laser, wrinkles

## Abstract

*Background and Objectives*: Photo/chrono-aging is usually expressed as facial discolouration, wrinkles, redness, elastosis, laxity, and dehydration, thus representing major signs of ageing that often lead to a negative phycological impact on a patient’s quality of life. Several types of treatment have been tested during the last decade, especially laser treatments. This article aims to share our experience in the treatment of photoaging with a new 675 nm laser source system on facial chrono-ageing. *Materials and Methods*: Thirty-five (35) patients were treated with the 675 nm laser device: 32 females (mean age 49 years) and 3 men (mean age 57 years), with Fitzpatrick skin types I–III (9% type I, 43% type II, 48% type III), facial wrinkles and hyperpigmented spots. The efficacy of this treatment was assessed using the Modified Fitzpatrick Wrinkles Scale (FWS), which was calculated before starting the treatment and after 6 months. The pain was evaluated using the VAS Pain Scale. *Results*: All 35 patients showed a significant improvement in facial wrinkles according to the FWS (from 1.96 to 1.73 at the 3-month follow-up, up to a value of 1.43 at 6 months). In a small group of patients, it was observed that 44% of them showed vascular moderate improvement and that 13% showed a vascular marked improvement after treatment. No side effects were detected except a mild erythematous rash in two patients, and the VAS Pain scale was assessed at 1.17. *Conclusions*: Red Touch allows a uniform and stable result to be achieved over time with minimum discomfort.

## 1. Introduction

Innovative physical principle-based technologies have been recently proposed to enhance the treatment of skin ageing. The potential of laser technology in skin care procedures is now being investigated [1]. Photo and chrono ageing affect the skin in both qualitative (wrinkles, discolouration, laxity, elastosis, atony, scarce superficial hydration) and quantitative terms (grade, extension, and the number of lesions); thus, aesthetic medicine aims to identify a treatment that could improve most of the visible signs of ageing. One of the most significant advancements in procedural dermatology over the past ten years is non-ablative laser resurfacing, which has replaced other treatments for a variety of aesthetic purposes. However, using them on people with dark skin types has raised safety concerns [2]. These lasers are less destructive than ablative lasers and “firm” the skin by stimulating the production of collagen in the dermis, while the epidermis is protected by the cooling system offered by the handpiece itself. The heat produced in the dermis causes the collagen to coagulate, which triggers the extracellular matrix and dermal collagen to be synthesised from scratch as part of the healing process. With the advancement of technologies, the side effects of these lasers, including scars and infections, have diminished over time. However, non-ablative lasers are less effective than ablative ones; thus, they are typically reserved for patients with mild to moderate photoaging [3,4,5,6].

From the literature, the 675 nm wavelength has been proven to be effective for resurfacing and pigmented disorders management [7,8,9,10]. Based on these scientific findings, the purpose of this research was to discuss our experiences using the 675 nm laser (RedTouch laser from Deka M.E.L.A, Calenzano, Italy) for the treatment of scars, wrinkles, and hyperpigmentation, using digital processing software for a more objective and accurate evaluation of the patient (in regard to tissue texture, vascularity, pigmentation, and laxity).

## 2. Materials and Methods

The study involved 35 patients who visited our centers specifically seeking a treatment that was intended to reduce the signs of ageing between January 2021 and July 2022. Of the 35 patients, 32 were women (aged between 35 and 65 years, with a mean age of 49 years), and 3 were men (aged between 49 and 63 years, with a mean age of 57 years) with Fitzpatrick skin type I–III (9% type I, 43% type II, 48% type III). The study’s exclusion criteria were as follows: the use of photosensitising drugs and/or the use of anticoagulant and/or immunosuppressive drugs; patients with light-triggered seizure disorders; pregnancy; patients with a personal or family history of skin cancer or with ongoing tattoos or skin conditions (e.g., eczema, etc.) in the areas to be treated; patients who had been exposed to the sun for several hours in the 3 weeks before treatment. All patients signed an informed consent form before starting the procedure. Photographic evaluation is a fundamental step in any comparative study, as it allows effective documentation and the comparison of the results acquired starting from the first session of the chosen therapeutic protocol. For an overall assessment, the images of patients were acquired with the patient in front, oblique and lateral positions (for both the right and left side of the face). In this case, digital photographs were taken of each patient according to the following scheme: at the first visit, after 3 months, and after 6 months. The photos were loaded into digital processing software (Vectra 3D software) for a better and more objective evaluation of the patient (in terms of tissue texture, vascularity, pigmentation, and laxity) and were subsequently saved in a database. Prior to each session, sufficient alcoholic and non-alcoholic facial cleansing was performed to clean the sebum, disinfect the skin, and mechanically exfoliate the stratum corneum. Based on the subject’s skin type and level of tolerance, a “test” region was used to determine the amount of energy needed to treat each patient. Within five to ten minutes, the test response was detectable. Mild erythema and some associated oedema were the endpoints. All techniques involved the use of an ultrasonic transmission gel and a transparent conductive gel. Every patient was photographed with the Vectra 3D (Canfield Scientific, Parsippany, NJ, USA) program at the beginning, following the first session, and just before and after the second treatment. The power, d-well time, and spacing for the device’s protocol were set at 10.0 W, 150 ms, and 1000 microns, respectively. The sessions were performed at an interval of 30 days. The treatment was performed by passing the handpiece over the affected areas without applying excessive pressure, with consecutive spots and no overlapping. Two laser passes were made. Areas close to the bone surface (forehead, cheekbone, etc.) were treated with a single pass to prevent the appearance of minor burns and/or hyperpigmentation. Topical anaesthesia was entirely optional; it was applied to only three patients and was completely removed before treatment. Following the treatment, the cooling of the skin was made with gauze soaked in cold water, and a soothing moisturiser was applied. Postoperative recommendations included using sunscreen with an SPF > 50 for the duration of the treatment and follow-up period. The effectiveness of this treatment was evaluated using the following procedures: (1) a comparison of digital photographs before and 3 and 6 months after the last treatment (a comparison was made between the photos acquired with and without the Vectra 3D software by an independent physician); (2) a 10-point visual analogue scale (VAS) (0, none; 1–2, mild pain; 3–6, moderate pain; 7–8, severe pain, 9–10, intolerable pain) to assess tolerance; (3) the Modified Fitzpatrick Wrinkles Scale (FWS) investigator-assessed from the baseline (0—no wrinkles, continuous skin line; 0.5—very superficial but visible wrinkle; 1—thin wrinkle; 1.5—wrinkles visible up to 1 mm deep; 2—wrinkles visible from 1 to 2 mm in depth; 2.5—wrinkle 2 to 3 mm deep; 3—deep wrinkles > 3 mm in depth were observed for all patients and each age group of patients (the following three age ranges were considered: 35–44; 45–54; 55–64). For all patients who showed erythema following the Vectra 3D software system, a vascular evaluation to assess vascular improvement after treatment was performed.

### Statistical Analysis

Paired Student’s *t*-test was used to test all of the outcome data for statistical significance with the SPSS program version 25.0 (IBM, Armonk, NY, USA). The level of significance for the statistical tests was (*p* < 0.01). Data were represented as the means ± standard deviation (SD).

## 3. Results

Based on a subjective clinical evaluation, 35 patients showed a significant improvement in facial ageing six months after the last treatment, with a visible improvement in laxity and atony. All patients experienced an improvement in skin texture, a noticeable reduction in chin-labial wrinkles (so-called marionette lines), and a re-definition of the mandibular area particularly. An increase in collagen production resulted in a tensor effect in the zygomatic region, which also resulted in a reduction in nasolabial folds. 

### 3.1. Wrinkles Evaluation

**Figure 1:**
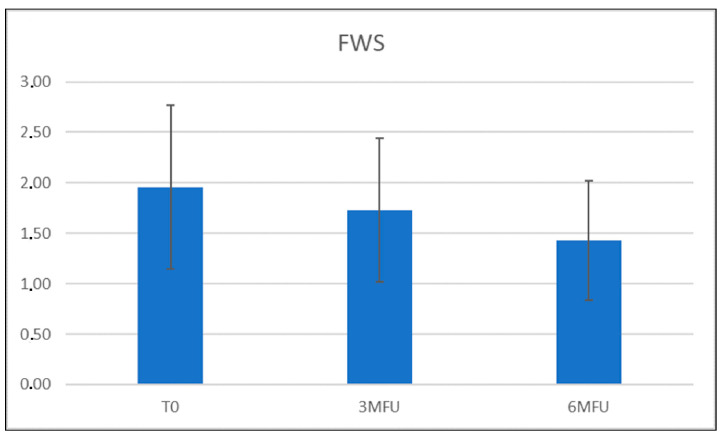
Graphical representation of Modified Fitzpatrick Wrinkles Scale (FWS) scores at baseline, 3 MFU, and 6 MFU. An improvement in wrinkles was observed in all patients.

**Figure 2:**
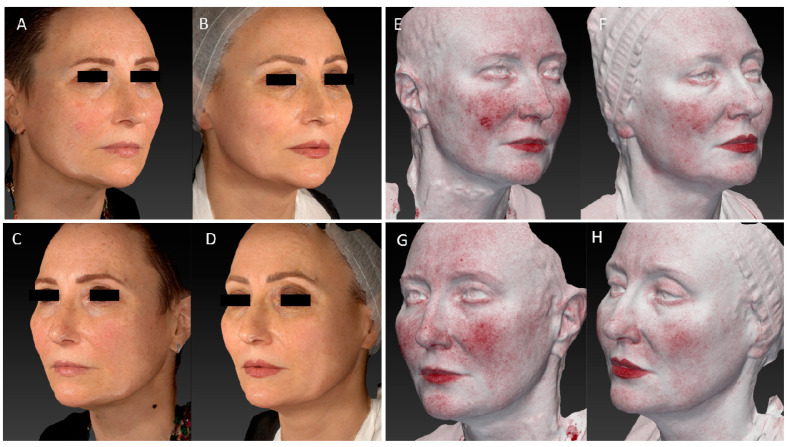
(**A**,**B**) Right lateral view of a female patient before and 6 MFU after laser treatment. (**C**,**D**) Left lateral view of a female patient before and 6 MFU after laser treatment. (**E**,**F**) Image of the right lateral view of the same female patient before and 6 MFU after laser treatment acquired with Vectra 3D software. (**G**,**H**) Image of the left lateral view of the same female patient before and 6 MFU after laser treatment acquired with Vectra 3D software. All images show an evident vascular and skin texture improvement in the whole patient’s face area. A tensor effect of the zygomatic region was obtained due to the increase in collagen production, which also led to a reduction in the nasolabial folds.

**Figure 3:**
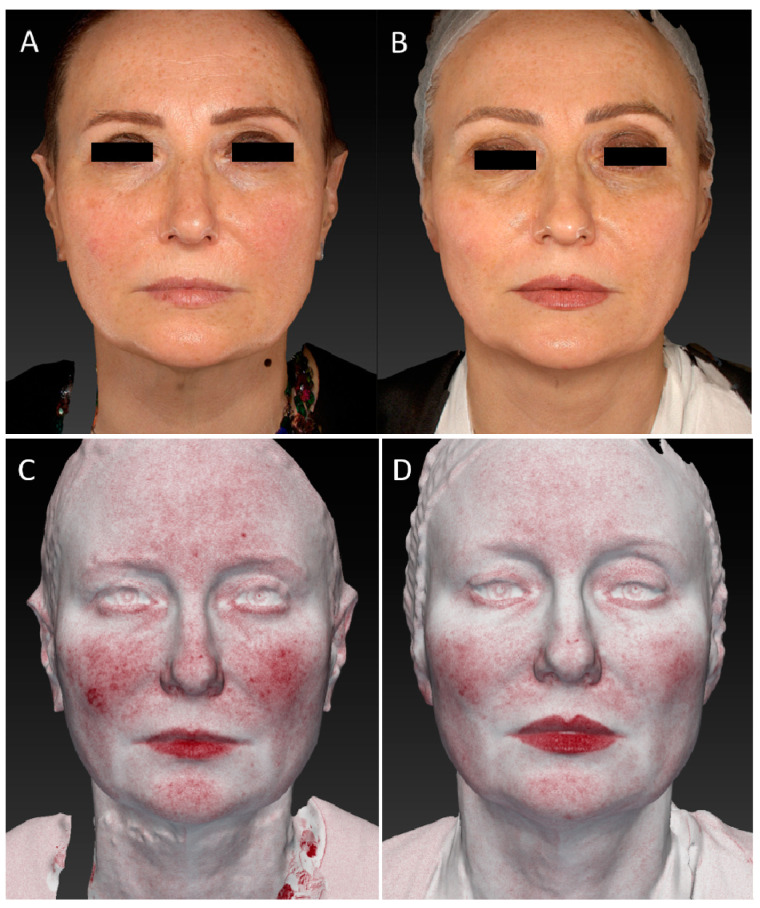
(**A**,**B**) Frontal view of a female patient before and 6 MFU after laser treatment. (**C**,**D**) Image of frontal view of the same female patient before and 6 MFU after laser treatment acquired with Vectra 3D software. All images show an evident vascular and skin texture improvement in the whole patient’s face area. A tensor effect of the zygomatic region was obtained due to the increase in collagen production, which also led to a reduction in the nasolabial folds.

**Figure 4:**
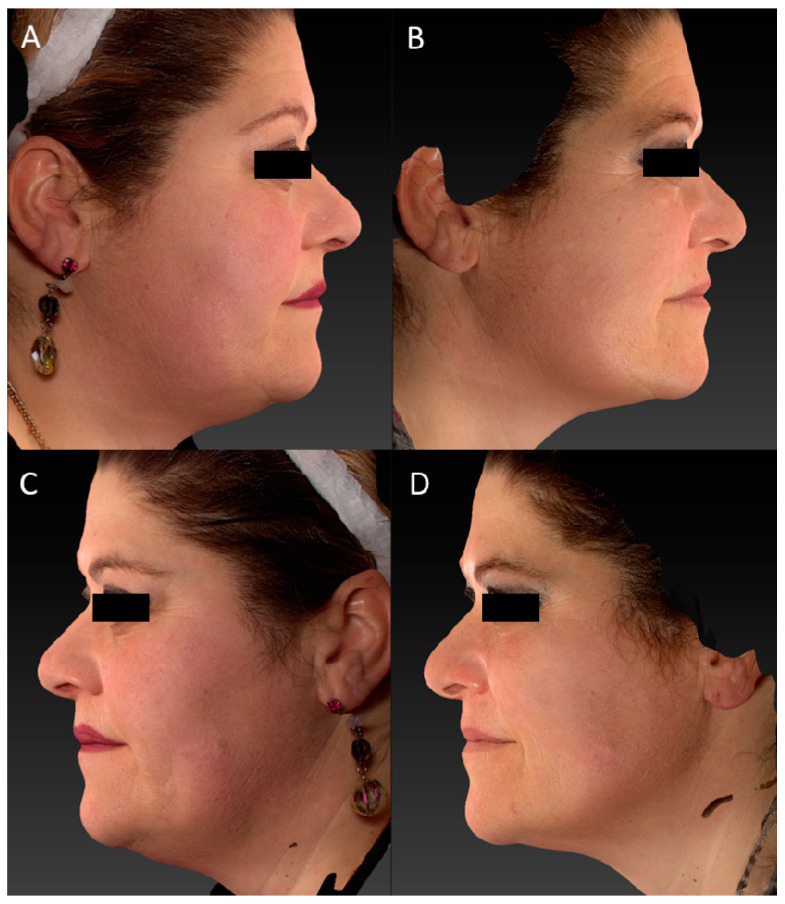
(**A**,**B**) Right lateral view of a female patient before and 6 MFU after laser treatment. (**C**,**D**) Left lateral view of a female patient before and 6 MFU after laser treatment. All images show a visible improvement in laxity, facial ageing, vascular and skin texture, and how a redefinition of the mandibular area was reached. A tensor effect of the zygomatic region was obtained due to the increase in collagen production, which also led to a reduction in the nasolabial folds.

**Figure 5:**
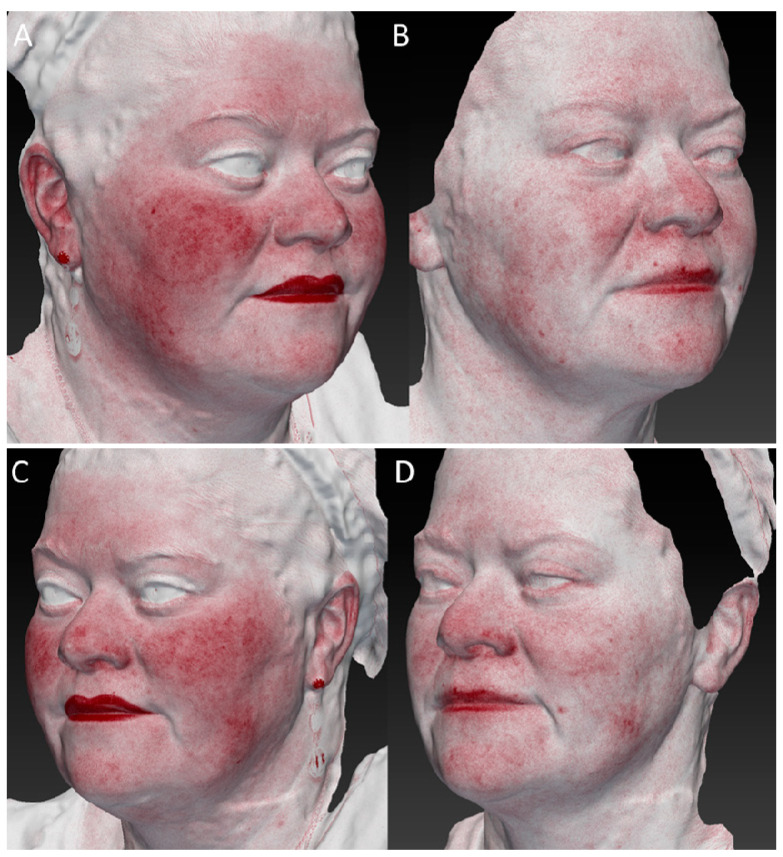
(**A**,**B**) Image of a right lateral view of female patient before and 6 MFU after laser treatment acquired with Vectra 3D software. (**C**,**D**) Image of a left lateral view of female patient before and 6 MFU after laser treatment acquired with Vectra 3D software. All images show a visible improvement in laxity, facial ageing, and vascular and skin texture, and how a redefinition of the mandibular area was reached. A tensor effect of the zygomatic region was obtained due to the increase in collagen production, which also led to a reduction in the nasolabial folds.

The improvement in wrinkles was consistent, as the scores decreased significantly from the baseline 1.96 (±0.81) on the FWS scale to 1.73 (±0.71) (*p* ˂ 0.01) at the 3-month follow-up after the last treatment and up to a value of 1.43 (±0.60) (*p* ˂ 0.01) at the last control (Figure 1). Due to its high affinity with melanin, a strong improvement in discolouration (hyperpigmentation and senile macules, i.e.,) was also observed on photographic evaluation (Figure 2, Figure 3, Figure 4 and Figure 5). 

### 3.2. Pain Assessment

Pain, measured by VAS, was minimal (Pain VAS: 1.17) for most subjects. 

### 3.3. Adverse Events

As an adverse effect, mild erythema was reported in 25% of the subjects (9/35), which arose at the end of the treatment and was self-resolved in about 2 h. 

### 3.4. Vascular Improvement

**Figure 6:**
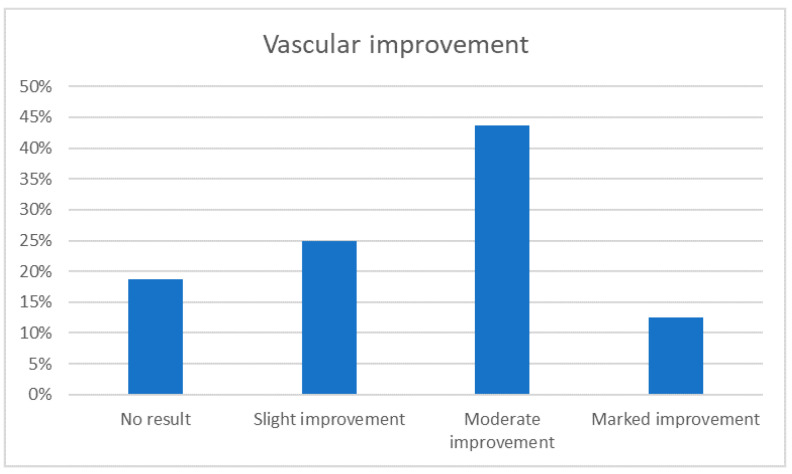
Graphical representation of vascular patient’s improvement.

Some patients examined with the Vectra 3D software and who presented with widespread redness were evaluated for vascular improvement. In total, 25% of them showed a slight improvement, 44% of them a moderate improvement, and a 13% marked improvement after treatment (as reported in Figure 6).

### 3.5. Wrinkles Assessment for Each Age Group

**Figure 7:**
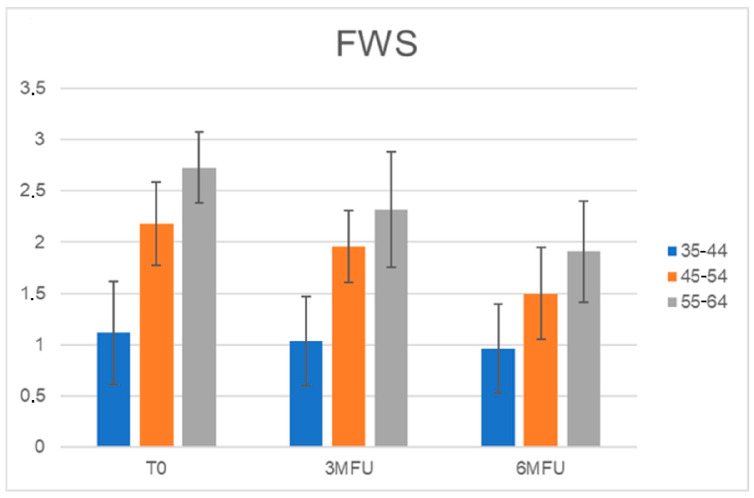
Histogram representation of FWS scale scores for each patient’s age group at baseline, 3 MFU, and 6 MFU. A more marked improvement in wrinkles was observed in the age group of older patients.

**Figure 8:**
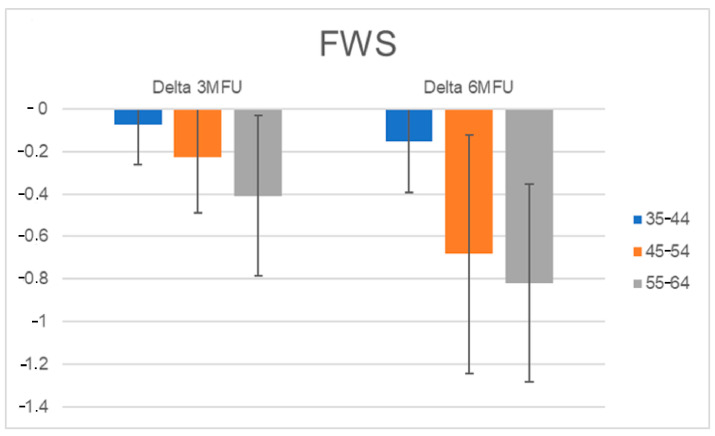
Histogram representation of Delta FWS scores calculated at 3 MFU and 6 MFU.

Furthermore, all patients were separated into three age groups (35–44; 45–54; 55–64), and the FWS scale scores were calculated for each age group. The improvement in wrinkles was more evident for the elderly population, and the scores decreased significantly from baseline 2.73 to 2.3 (*p* ˂ 0.01) at the 3-month follow-up after the last treatment, up to a value of 1.9 (*p* ˂ 0.01) at the last control (Figure 7 and Figure 8).

## 4. Discussion

The study device was based on the emission of red light at a wavelength of 675 nm through a 15 × 15 mm scanning system that could cause selective thermal damage to the skin that penetrated the dermis on average at a depth of more than 1 mm. Several characteristics could be used to control the energy provided (power, pulse duration, and distance between the microthermal zones). Furthermore, a 5 °C contact skin cooling mechanism was included in the study system to shield the epidermis from heat-related damage. The heat impulse generated at this level denaturised the collagen fibres, which led to the synthesis of new collagen. This wavelength showed high selectivity with collagen. Type I and III collagen play relatively significant roles in the formation of collagen in healthy human skin, with respective contributions of 80–85% and 10–15% [11]. Currently, at least fourteen different types of collagens are known [12]. Among them, they play a crucial role during the formation of skin, and their content fluctuations and relationship have been observed during ageing. In particular, type I collagen makes an essential contribution to the skin concerning thickness, while type III collagen shows a greater involvement in establishing the reticular structure of the skin [13]. The relationship between the type I and type III collagen content was found to increase with age, as demonstrated by Qiu and colleagues [14,15], who found that the type I and III collagen content was higher in younger age groups and lower in the elderly.

These findings regarding age-dependent stages of collagen types and skin recovery could be used to prevent hypertrophic scarring and pre-rejuvenation of the skin [16]. In vivo and in vitro investigations demonstrated the therapeutic effects of near-infrared light (NIR) and Red-light, including photo-rejuvenation [17], hair regrowth, and a healing effect [18]. The fibroblasts’ proliferative response and wound-healing capabilities of the 636 nm laser could control oxidative stress [19,20]. Lasers with wavelengths between 524 and 904 nm have been demonstrated in several wound models to speed up the wound healing process, boost collagen synthesis, enhance epithelial differentiation, and stimulate dermal vascularity [21,22].

In the rat model, the use of 685 nm, when applying a dose of 20 J/cm^2^, stimulated collagen deposition, an increase in myofibroblast cells, and improved the reorganisation of healed tissue [23]. Several studies conducted on cultured fibroblasts revealed that 860 nm stimulated cellular proliferation [24], 812 nm increased DNA synthesis [25], 660 nm up regulated the release of the basic fibroblastic growth factor [26], and 632.8 nm induced the activation of fibroblasts into myofibroblasts [27]. Moore and colleagues [28] showed that 665 and 675 nm stimulated a faster proliferation in fibroblasts compared to endothelial cells, whereas 810 nm light inhibited this process. These different outcomes could be caused by a variety of variables, such as the laser’s irradiation parameters (such as wavelength, power density, and fluence), the cells that were exposed to the laser, or an underlying wound-healing deficiency in the in vivo system. 

By enhancing the expression of heat shock protein and collagen types I and III at the cellular, histologic, and therapeutic levels, light-based therapies have been shown to influence and possibly reverse skin ageing. Premature ageing and wrinkles can be caused by modifications to the typical collagen type I/III ratio in the face skin. By altering the cell transcriptome through a variety of different gene expression mechanisms, visible and NIR light can affect differentiation, proliferation, and collagen synthesis [17]. Near-infrared light (780–1000 nm) and red light (600–760 nm) have an impact on several processes in living cells and tissues. In particular, the 675 nm wavelength shows a high affinity for collagen fibres and melanin [29] with minimal interaction with haemoglobin. This new device, with a wavelength of 675 nm, acts directly on the collagen component based on its spectral absorbance. This is in contrast to laser systems that use wavelengths below 650 nm, which are highly absorbed by haemoglobin, and wavelengths above 950 nm, which are primarily absorbed by water.

This way, the heat was transferred directly to the collagen fibres without hitting other chromophores. The heat was instead diffused to the surrounding areas, allowing the immediate shrinkage and denaturation of the collagen as well as subsequent new collagen production via fibroblast activation. In a recently published study, the use of the 390 J/cm^2^ of 675 nm laser wavelength was shown to be effective in stimulating type III collagen synthesis in human cultured fibroblasts while not affecting cell viability or proliferation, confirming its anti-ageing effect in the aesthetic field [30].

Due to its high affinity to collagen, a 675 nm laser device has also been suggested for the treatment of other collagen-rich lesions, like acne scars, with good outcomes [4]. As demonstrated by a histological examination performed on a patient, the energy supplied to the collagen induces collagen regeneration, favouring dermal collagen production and the rearrangement of elastic fibres [8]. Since it has a high affinity for melanin, the subject device was recommended for the treatment of the hyperpigmentation condition, as mentioned in the Introduction section [3,4,5,9]. Our study focused on the effectiveness of a single anti-ageing treatment in the medium term in a cohort of 35 patients who presented to our clinics with the same request: to counteract the effects of facial skin ageing. Our findings also focused on the effect of the 675 nm wavelength on collagen stimulation in young patients. For this reason, the study population was divided by age group, and an evaluation of the wrinkles, which was carried out, revealed an overall improvement for all the groups. Although a more marked improvement was observed in the older population, an effect was also observed in the younger population, suggesting the potential use of the 675 nm laser device in the prevention of ageing as a “prejuvenation” treatment [31]. In addition, the patient’s vascular improvement was observed. Although haemoglobin is not the primary target, the capacity of 675 nm to interact with the collagen of blood vessels results in an increase in the dermal tone and turgor as well as the higher continuity of the vascular system, as shown by a high percentage of patients with moderate vascular improvement. The treatment was straightforward, non-invasive, and had few side effects (the most common side effect was mild self-healing erythema that was resolved in about 2 h). Because of the preventive cooling of the skin, the procedure was also relatively painless. The absence of microscopic necrotic epidermal debris or dermo-epidermal detachment, which is typical of the postoperative course of NIR systems, was most likely due to the increased focus on small spots (100–300 µm). The absence of scabs and/or micro-crusts after treatment had a minor impact on the patient’s interpersonal relationships. 

RedTouch is a promising device for the treatment of photo-ageing, chrono-ageing, and pigmentation dysfunction owing to its capacity to act on melanin and collagen fibres. All these observations justify the already excellent results experienced by our patients. In 2020, we preliminarily tested this device on 29 patients (19 women and 10 men), with an average age of 52 years (range 47–66), who were treated with two sessions 30 days apart. We reported excellent results and reported data on the efficacy of laser resurfacing at 675 nm [6]. The non-ablative and ablative (CO_2_ or infrared Er:YAG) devices with a 1320–1540 nm spectrum emission that are currently available target water to transform laser energy into heat. The photothermic effect then generates an increase in the temperature that indirectly involves and stimulates collagen fibres. Although effective, these types of treatment require significant downtime to be managed and concorded with the patient. On the contrary, the RedTouch System works directly on the collagen component of the skin: the selectivity of its emission allows it to act within an optical window that maximises its affinity with collagen fibres and minimises interactions with the vascular component. This mechanism of action translates into a procedure that uses minimal energy levels to facilitate the execution of the treatment and does not require the special preparation of the skin at the same time. In the current study, we focused on a more structured evaluation of the results with the aim of objectively reporting on pain, the improvement of wrinkles, and the safety of this laser device. For this reason, we decided to evaluate the patients through a scale (FWS) and a strict photography comparison. Finally, for all patients showing erythema after the Vectra 3D software system measurement, a vascular evaluation to assess vascular improvement post-treatment was performed.

Our future goal is to increase the number of patients and evaluate the long-term effects of laser effects.

### Limitations of the Study

Limitations include a limited number of patients, a lack of evaluation of the long-term effects of this procedure, and a lack of patient satisfaction questionnaires.

## 5. Conclusions

In conclusion, this study makes a significant contribution to the efficacy of the study device. The study device allowed a uniform and stable result to be achieved over time with minimum patient discomfort. Compared to the systems currently on the market, such as radiofrequency, high-intensity focused ultrasound (HIFU), or Microneedling, RedTouch acts directly on the collagen in the dermal layer. The 675 nm laser treatment, compared to other techniques like surgery, is painless (no anaesthesia required), easy to use, and requires short sessions that lead to faster patient recovery and side effect-free procedures. Furthermore, although the wavelength used has a low affinity for haemoglobin, it can enhance vascular appearance by acting on the collagen fibre levels in the vessel wall, increasing their elasticity.

## Data Availability

The data that support the findings of this study are available from the corresponding author upon reasonable request.
